# Doctor–Patient Communication in Primary Health Care: A Mixed-Method Study in Fiji

**DOI:** 10.3390/ijerph18147548

**Published:** 2021-07-15

**Authors:** Swastika Chandra, Masoud Mohammadnezhad

**Affiliations:** Department of Public Health, School of Public Health and Primary Care, Public Health Campus, Fiji National University, Suva 7222, Fiji; chandrasuzy12@gmail.com

**Keywords:** doctor–patient communication, communication behaviour, patient expectation, mixed-method study, Fiji

## Abstract

From a clinical perspective, effective and efficient communication is part of a strategy to ensure doctors are providing high-quality care to their patients. Despite the positive impact of effective doctor–patient communication on health outcomes, limited information is available on this in Fiji. This study was carried out to determine the current patients’ perception of doctors’ communication behaviour and identify factors affecting the doctor–patient communication in Fiji. This mixed-method study was conducted in the outpatient setting of three randomly selected health centres in the Suva Subdivision, Fiji. For the quantitative phase, systematic random sampling was used to select the 375 participants who completed the structured questionnaire; of those, 20 participants were selected for the qualitative interview. From the patients’ perception, 45.6% of them perceived doctors’ communication behaviour as good, 53.6% as fair, and 0.8% as poor communication behaviour. Qualitative findings highlight factors such as the attitude of the doctors, their approach, their interaction with the patients, and them providing an explanation as important factors during doctor–patient communication. In Fiji, the majority of patients perceived doctors’ communication behaviour as fair to good and the doctors’ skills were important for effective doctor–patient communication. This study highlighted the importance of doctor–patient communication and suggested that doctors might not be practicing patient-centred care and communication; thus, they need to upgrade their patient-centred communication skills.

## 1. Introduction

There is nothing new or innovative in stating that doctor–patient communication is critically important. Indeed, there is a pool of literature detailing doctor–patient communication, its impact on health outcomes, and highlighting areas for improvement [1,2,3,4,5,6,7]. However, for Fiji, which is a developing and multicultural island in the Pacific, there is little or no data on current perceptions of doctor–patient communication. Additionally, current studies have not been carried out in similar settings to Fiji. Therefore, it will be difficult to advocate for changes in policy or practice for improvement in health care services as it is not known what is effective and what needs to change. This paper is an attempt to respond to this gap in the literature in the Fijian health care system setting.

Communication is a process involving the transmission of information or exchange of ideas between two people, the communicator and the recipient. Communication skills refer to, ‘the ability to convey information to another effectively and efficiently’ [1], including nonverbal and verbal interaction, voice tones, phrases, gestures, facial expressions, and body language that one uses while interacting with another person [2]. Communication and interpersonal skills are included in the six areas in which doctors in training need to demonstrate competence, as identified by the Accreditation Council for Graduate Medical Education (ACGME) [8]. A doctor’s communication behaviour during a consultation is as important as the information being conveyed to the patient [9].

The important aspects of doctor–patient communication includes developing a good interpersonal relationship, being a listener, exchanging information, and making patient-centred management plans [3,4]. Good and effective doctor–patient communication improves patients’ trust in their doctors and their satisfaction level and indirectly affects patients’ health outcome, such as improvement in symptoms and adherence to medical treatment [5,10,11,12]. Hence, from a clinical perspective, effective and efficient communication is a part of a strategy to ensure that doctors are providing high-quality care to their patients [1,6,7].

In primary care, patients strongly prefer a patient-centred approach with communication, partnership, and health education [13]. In contemporary times, doctor–patient consultation is more patient centred, involving patient-centred communication and care [14]. Patient-centred care simply means being more responsive to patient perspectives and needs, perspectives such as their concerns, expectations, ideas, and feelings, with patient values guiding decision making [14]. It means that the doctor is being receptive to the patient’s needs through incorporating the patient’s perspective and experiences in making a management plan [15]. Patient-centred communication builds on discussions and decisions that involve sharing of information, providing empowering and compassionate care, being sensitive to patient needs, and building relationships [16].

To achieve patient-centred care, patient-centred communication helps in creating opportunities and supports patients in becoming active participants in their health care leading to effective doctor–patient communication [13,17,18]. Some of the ingredients for patient-centred communication include the doctor’s listening skills, empathy, the patient’s storytelling skills, a nondistracting environment, medical expertise, and time. While conversing, doctors should try not to sound complicated, should avoid the use of medical jargon, use some repetition and check that the patient understood what was being conveyed to them [15]. Improved communication with patients in routine patient care will lead to better subjective health outcomes and higher patient satisfaction, without extra expenses [18].

Good doctor–patient communication is more likely to result in patients being satisfied with their care, follow the doctor’s advice, adhere to their treatment plan, better mental health, enhanced psychological adjustments, especially with poor prognosis and delivery of bad news, and a decrease in the number of hospital admission days [18,19]. These lead to decreases in recurrent visits and admission with a reduction in overall economic costs. Better communication can also result in better doctor satisfaction in terms of fewer patient complaints, better job satisfaction, less work-related stress, and reduced burnout [19]. Hence, in the effort to improve the quality of health care, the evaluation of doctors’ communication skills is increasing [20]. These evaluations are usually carried out through patient experience surveys and external raters, although patient surveys can be used alone also, with a study showing that there is a moderate correlation between patient and external rater scores [20].

In Fiji, medical services are mostly provided by the government health centres, and they provide free services to patients. Due to the high number of patients attending health centres and also limited available health facilities, patients have to wait a long time to visit a doctor. Patients attend health centre and refer to a doctor that is available. Doctors access patients’ information through a health information system that records all the patients’ medical history. In Fiji, the Health Ministry has voiced concerns over poor communication of doctors which has resulted in complaints against doctors, leading to doctors being disciplined due to poor communication and decision making [21]. An apparently ‘simple’ measure, such as improving the communication skills of doctors, can be of long-term health and economic benefits, as mentioned earlier. Therefore, for income-constrained countries such as Fiji, health policymakers need to focus policy and practice efforts on assessing and improving communication skills. Hence, this study was undertaken to respond to this need in Fiji by assessing and understanding the factors influencing the patients’ perception of communication behaviour of doctors.

## 2. Methodology

### 2.1. Study Setting and Design

This was a mixed-method study with a quantitative and qualitative design, conducted in outpatient departments of randomly selected health centres in the Suva Subdivision of Fiji (from July to August 2018). A cross-sectional survey of patients was undertaken, followed by semistructured interviews with a subset of the larger sample. In this study, the survey and qualitative study were conducted simultaneously. A qualitative approach with in-depth interviews was carried out to support the findings from the quantitative study. The concept of methodological triangulation was used whereby the quantitative design aided in providing a validated method to measure patient perceptions of doctor’s communication behaviour, whereas the qualitative design aided in gathering an in-depth understanding of patients’ experiences and expectations of communication with doctors [22,23,24].

### 2.2. Study Participants

This study included patients aged over 18 years, of any ethnic background, of any gender, with self-identified Fijians who understood either English, Hindi, or Fijian. Those who were not willing to participate or had some form of cognitive disorder were excluded from the study. Written consent forms were given to those who met the inclusion and exclusion criteria and upon return, respondents were recruited into the study.

### 2.3. Sampling and Sample Size

There are seven health centres in the Suva Subdivision, and from this, three health centres were randomly picked from a jar of chits with the health centre name. For the cross-sectional survey, participants were selected using systematic random sampling whereby every third person waiting to see the doctor was approached and those who met the inclusion criteria and were willing to participate were selected for the study.

The estimated sample size for the quantitative phase was estimated using 50% response distribution, 5% margin of error, and 95% confidence interval [25]. The estimated sample size was 377, and in consideration of nonresponse of 10%, the estimated sample size was 410 participants. Participants were approached while waiting to be examined by the doctor, and those who met the inclusion criteria were given the questionnaire. Some filled it based on past consultation, while some filled it after the same-day consultation.

For the qualitative phase, convenience sampling was used. The participants from the cross-sectional survey were approached at the same time and those interested (by signing the consent form) in taking part in the interview were selected at the same health centre. The total number of participants for the qualitative phase was 20 based on data saturation. Guest et al. proposed that the minimal sample size needed to obtain enough information is often around 12 participants in homogeneous groups [26].

### 2.4. Data Collection Tool

The cross-sectional survey was conducted with a structured questionnaire with close-ended questions. The questionnaire was guided by the concept of the ‘four-habits model’ for medical interviews; invest in the beginning, elicit the patients’ perspective, demonstrate empathy and invest in the end [27]. In a review of the literature concerning doctor–patient communication, the important aspects of doctor–patient communication includes developing a good interpersonal relationship, being a listener, exchanging information, and making patient-centred management plans [4]. The questionnaire comprised statements and questions focusing on the above habits and skills which were extracted from questionnaires that have already been used and validated in previous studies [28,29].

It was ensured that the statements and questions extracted had high internal reliability with a Cronbach’s score of 0.7 or more [28,29]. Two experts in doctor–patient communication in Fiji were given the questionnaire for content validity, with minor changes carried out after the review. For face validity, the printed questionnaire was given to 10 patients attending the outpatient department (these patients were excluded from the final study) to assess whether it was clear, legible, simple, and easy to understand. The majority of patients understood the statements in the questionnaire. They filled the form with ease and returned it within 10 min; therefore, no changes were made to it. The variables including sex, age, ethnicity, employment status, educational level, number of visits to the health centre, and communication behaviour of doctors were used during the analysis.

The questionnaire contained two components—component 1 focused on the socio-demographic information of the participant, and component 2 contained 8 close-ended questions which focused on the communication behaviour of the doctors. Response to the statements and question utilized a Likert 5-point scoring scale; strongly agree, agree, not sure, disagree, and strongly disagree. For positive statements such as, ‘My doctor gave me the opportunity to ask questions’, strongly agree was given 5 points and regressed down to 1 point for strongly disagree for positive statement. For negative statements such as, ‘Sometimes I feel the doctor ignores my concern’, strongly agree was given 1 point, and strongly disagree was given 5 points. The maximum score could be 40, while the minimum score could be 8. With the use of bilingual translators, the questionnaire was translated into two other languages, Fijian and Hindi. To ensure that the contents of the original questionnaire matched the translated version, the translated versions were translated back from Hindi and Fijian to English by different translators [30].

Although the reliability of the questionnaire was not tested before being used for the study, the reliability was tested (using Cronbach’s alpha) using the data collected during this study (375 questionnaires). For component 2, Cronbach’s alpha score was 0.76 for the 8 items.

For the qualitative phase, semi-structured interviews were undertaken, which took 30–40 min long per interview. All interviews were audio-recorded. The interview was facilitated with the use of open-ended questions which focused on the participant’s feelings and opinions about their interaction with their doctors and expectation of doctors’ behaviour during the consultation. Questions focused on issues related to their medical consultation overall and communication such as, ‘Were you able to understand what all the doctor advised you? Please explain’, ‘Did you feel scared, relaxed, comfortable or safe while with the doctor? Please explain why’.

### 2.5. Data Analysis

The raw data from the quantitative questionnaire were entered into Excel and after data were cleaned, it was transferred into IBM SPSS, version 21 (IBM Corp, Armonk, NY, USA). The sociodemographic variables and communication behaviour were analysed using descriptive analysis (mean, standard deviation, and frequency). For analysis, communication behaviour was divided into 3 categories: ‘Good communication behaviour (32–40 points)’, ‘Fair communication behaviour (17–31 points)’ and ‘Poor communication behaviour (8–16 points)’. The category was based on the scoring point range and standard deviation (SD); scores more than or equal to +1 SD were taken as good communication or full trust, scores less than or equal to −1 SD were taken as poor communication or lack of trust, and scores between ±1 SD were taken as fair communication or partial trust.

For all the interviews, the audio recorded information was transcribed into a Word document using modified verbatim transcription, after the direct translation was carried out for a few interviews. Audio recordings were listened to three times in order to transcribe the data accurately. Few transcribed interviews were randomly chosen and checked for accuracy by a co-researcher. These data were then analysed using manual thematic analysis [31]. The 6-phase guide framework by Braun and Clarke was used for thematic analysis [28]. Initial coding was performed for the data, and after reviewing this list, some codes were revised while some were added. The emerged codes and themes were discussed between the main researcher and co-researchers to make sure they were identified correctly. These coded statements were then grouped together whereby common themes were identified. Themes related to patient satisfaction and its determinants were prioritised.

### 2.6. Ethic Consideration

Ethics approval was obtained from the Fiji National University College Health Research Ethics Committee (CHREC) and from the Fiji National Research Ethics Review Committee (FNRERC) in June 2018 with ethical approval number 2018.126.C.D. After both the ethics committee’s approval was received, approval was requested from the subdivisional officer and written requests and approvals were obtained from the medical officers in charge of the selected health centres.

## 3. Results

### 3.1. Quantitative Study

#### 3.1.1. Demographic Characteristics of Participants

A total of 375 completed questionnaires were returned from the 410 questionnaires distributed, hence a response rate of 91%. Data from these questionnaires were analysed, and Table 1 summarises the sociodemographic characteristics of the participants. The mean age of the participants was 38 years (SD = ±15) with a minimum age of 18 years and maximum age of 85 years. The majority of participants were female (61.1%) and of i-Taukei (54.9%) ethnicity, followed by Indo-Fijian (37.8%) and Others (7.5%). Most of the participants gained either secondary or tertiary level education (91.5%). Around half of the participants were employed (57.6%), while 19.2% were doing domestic duties, 13.3% were studying, and 9.9% were unemployed.

#### 3.1.2. Participants Response to Communication Related Statements

There were eight statements used to assess doctors’ communication behaviour, as summarised in Table 2. Although all statements are assessing the doctor–patient communication, statements 3, 4, and 6 are more specific about patient-centred communication. The majority of participants agreed that overall doctors treated them in a way that made them comfortable (85.1%), clearly explained to them their medical condition (81.1%), checked to ensure they understood everything (77.6%), provided them as much information as they wanted (71.7%), and gave them an opportunity to ask questions (70.1%). Almost two-thirds of the participants overall disagreed that the doctors ignored their concern (62.7%), while 23.3% felt that the doctor did ignore their concern. Again, around two-thirds of the participants overall disagreed that doctors used too many technical terms that confused them (65.9%), while 20% agreed that the doctors used too many technical terms.

#### 3.1.3. Levels of Communication

Communication behaviour scores were converted into a categorical variable; divided as ‘Good communication behaviour (32–40 points)’, ‘Fair communication behaviour (17–31 points)’, and ‘Poor communication behaviour (8–16 points)’, as summarised in Table 3. For the patient’s perception of the doctor’s communication behaviour during the consultation, 45.6% perceived good communication behaviour, more than half (53.6%) as fair communication behaviour, and 0.8% as poor communication behaviour. The mean score for communication behaviour was 30 points (±5), with a minimum score of 16 points and a maximum score of 40 points. Higher scores generally represent good and more patient-centred communication.

### 3.2. Qualitative Study

#### 3.2.1. Demographic Characteristics of Participants

The qualitative data were analysed using manual thematic analysis. A total of 20 interviews were conducted, of which there were an equal number of males and females (50%) and an equal number of i-Taukei and Indo-Fijians (50%). The mean age of the participants was 42 years (SD = ±12), with ages ranging between 23 years to 66 years. There were 14 participants who were below 50 years of age, and 6 participants who were 50 years or older. Table 4 summarises the characteristics of the participants.

After the analysis of the data, the themes generated included the characteristics of the doctor’s communication behaviour, patient-centred communication, and patient’s expectation during consultation.

#### 3.2.2. Characteristics of Doctor’s Communication Behaviour

For the majority of participants, the overall attitude of doctors matters during their consultation, including the doctors’ tone and approach upon entering the consultation room. Participants appreciated doctors for being polite and friendly, which made them feel comfortable.

‘The doctor was friendly and polite’

(49 years, i-Taukei male).

‘I feel comfortable, they are not harsh or rough with you… they do their part’

(59 years, i-Taukei male).

For a few, the doctors’ approach decreased their frustration with a long wait and their nervousness.

‘In the beginning, I am bit scared but when I enter the room and the way the doctor greets me, I can understand that I can be comfortable with this doctor’

(31 years, Indo-Fijian male).

The first impression of the doctor (through their verbal and nonverbal interactions) also made a difference to participants. Some patients appreciated the way they were welcomed by the doctors; their facial expressions, a simple smile, and greeting, which made them feel welcome and comfortable.

‘The ways doctors approach me…uhm and the way they talk and their tone’

(23 years, Indo-Fijian male).

‘Their first expression… the way they look and greet…’

(42 years, i-Taukei male).

Participants even felt that some doctors made extra effort to make them feel comfortable during the consultation simply by looking at them as a person and not simply another patient in the queue.

‘I’ll say probably he had treated me as a person…he left his level of being a doctor and explained to me as a lay person, cared for me…I was comfortable’

(30 years, Indo-Fijian male).

While the majority of participants had appreciated the doctor’s attitude in a positive way, a few experienced the opposite from their doctors. The doctor was rude to them and did not talk or explain things in a kind or respectful manner.

‘…the doctor was rude, just saw, wrote the prescription…just sitting and didn’t ask’

(38 years, Indo-Fijian female).

‘…but at times might not be all the information that I needed. Sometimes the doctors don’t explain well, depends on the doctors…’

(66 years, Indo-Fijian male).

Providing explanations and ensuring that patients understood the advice or treatment plan was important for almost all the participants. Whether it was primarily related to their medical condition, dietary advice, or treatment plans, participants stated they were satisfied with the consultation and treatment plans.

‘They explained things to me… they discuss the treatment with me, explained the side effects and asked if I was allergic to any medicine’

(51 years, i-Taukei female).

‘The 100% is how they perform when they explain the sickness to you, the right medication and what it can do and the side effects’

(59 years, i-Taukei male).

While for a few, they got more frustrated and even angry when the doctor did not talk to them respectfully or explain things appropriately.

‘Felt angry as doctor didn’t explain or examine… couldn’t ask because of fear… looked bit stubborn doctor’

(39 years, Indo-Fijian female).

#### 3.2.3. Patient-Centred Communication and Care

Some participants talked about their experiences which reflected ‘patient-centred communication and care’ and these participants appreciated their involvement in the discussion. They appreciated the fact that the doctors were listening and respecting their opinion and this made them share more information with the doctor.

‘They respect your opinion… they gave me time to ask the questions and I asked them more questions’

(51 years, i-Taukei female).

Allowing patients to ask questions or clear their doubts or have a say in their management was one of the positive feedback from the participants, and some doctors involved them when deciding the treatment.

‘Here they listen to my opinion’ (44 years, Indo-Fijian male), while another 39 years, Indo-Fijian female stated, ‘The doctors talk very nicely, they let me ask questions…’.

‘Yes, they discuss the treatment with me, explained the side effects and asked if I was allergic to any medicine’

(51 years, i-Taukei female).

Some participants stated that they were not fully involved during the consultation process and faced a one-way communication whereby the doctor was speaking and making the majority decision. Though, they mentioned that it would have been appreciated if the communication was more collaborative involving two-way communication whereby patients were equally involved in the conversation. In addition, some participants thought it was difficult to share all information with the nurses, which they would rather share with the doctors and if doctors do not conduct a proper medical interview, they might end up with inadequate management plans.

‘…most questions have been asked by the nurse, not by doctor. Nurse just gives, and doctor reads it. She can read and ask again, it’s a follow up questions… sometimes you can say different thing to nurse’

(43 years, i-Taukei male).

A few participants also showed disappointment as a result of inadequate relaying of information to the patient on the management plans.

‘The medicine was not here, and I have to buy the medicine. He (the doctor) never ask and told that I need to buy it. I would appreciate if the doctor can ask’

(30 years, i-Taukei male).

In contrast to the doctors taking the lead in initiating better communication, some participants highlighted that the patients need to be responsible and play an active role rather than being passive recipients.

‘If you ask questions, then they can give you answers. If you don’t ask, then they don’t know what is happening. If you keep quiet…they just write and give prescription. They listen when I ask. Have to ask if don’t understand’

(35 years, i-Taukei male).

#### 3.2.4. Patients’ Expectations—Ideal Doctor–Patient Communication

Participants expected their doctors to consult them in a certain manner; either ask questions, examine them or explain things to them.

‘…well I was expecting for her to give me a medical check like I would prefer if she (the doctor) could examine me but she didn’t’

(43 years, i-Taukei male).

When participants came to the hospital, they wanted to know what was wrong with them and how they could recover.

‘I wanted to know what was wrong with her, but the doctor didn’t explain anything’

(39 years, Indo-Fijian female).

Participants believe that if doctors can spend more time per patient, it will result in better consultation.

‘I hope we can spend more time, average of five minutes at the moment’

(51 years, i-Taukei female).

Despite the workload, the participants at least expect doctors to be friendly and initiate a conversation with proper history taking, examination, and explanation to them.

‘Some doctors just want to go fast as possible whereas they should be friendly and ask about the problem and explain what’s wrong with the patient’

(25 years, Indo-Fijian male).

‘Some of the doctors are rude and I understand that its part of their work…’

(30 years, Indo-Fijian male).

## 4. Discussion

The study findings indicate that for a developing, middle-income Pacific Island country, around half of patients attending outpatient services in the Suva Subdivision in Fiji, consider doctors’ communication behaviour as fair rather than good (53.6% vs. 45.6%). In contrast, a study in Chicago (a city in a developed, high-income country) found that the majority of the participants perceived the doctors’ behaviour as either excellent or very good (equivalent to good in this study) [32], highlighting the relative subperceptions of communication in Fiji. The results of this study also showed that the majority of participants agreed doctors treated them well, explained their medical condition properly, or provided them as much information as they wanted. This might be due to the characteristics of participants as the majority of them had higher education. They were able to ask their questions better and know more about their health condition so it could affect their perception towards doctors’ communication.

Our study findings suggest that doctors are currently practicing both one-way and two-way communication styles, although it generally seems that doctors play a dominant role during consultations, as evident from statements 3, 4, and 6 of the qualitative results. Although the majority of participants overall agreed with these statements, these three statements had the lowest percentage of participants agreeing with them, compared to other statements.

This was further supported by qualitative data, according to which some participants felt they were able to share their opinions and queries with their doctors, while others felt that they were not empowered to engage with their doctors. The important factors that made patients comfortable were the attitude of the doctors, the approach of the doctors when patients arrived at the consultation, involving the patients in discussions, and providing advice, explanations, and prognoses in nonscientific language. Sometimes, due to the difference between patients’ expectations and the actual encounter, doctors are faced with difficult encounters and patients end up dissatisfied [33]. The qualitative findings also suggested that some doctors currently practice patient-centred communication and care, while others do not, leading to feelings of inequity or unfairness. It would be recommended for the subdivisional supervisors to organise workshops to train, refresh, and highlight the importance of the communication skills of all the doctors, including patient-centred communication and care. These findings might guide the relevant undergraduate and postgraduate programs’ teaching curriculum with the incorporation of communication skills and patient-centred communication and care as a major topic.

Doctors’ interpersonal skills such as greeting patients, being polite, friendly, and respectful are appreciated by patients, resulting in patients being satisfied with their consultation and treatment provided. Advocating health literacy by sharing medical information, advice, and treatment plans also results in patient satisfaction, whereas those who had a very limited conversation with their doctors with no explanation or advice given were disappointed. This finding is consistent with the findings from other studies [34,35,36]. Findings from a study in Pakistan showed that several components of doctor–patient interaction, such as technical expertise, time spent, general approach and communication, and alternative health care were associated with patient experiences of vulnerability [34]. Similarly, a study in Singapore identified that most of the complaints lodged concerning health care were communication issues, including specific nonverbal and verbal communication errors, content errors, and poor attitudes during doctor–patient interaction [35]. In terms of doctors providing explanations during their interactions with patients, one study found that patients were more likely to practice good self-management when physicians provided sickness-related and management-related information in such a way that patients completely understood the importance of self-management [37]. Given that Fiji’s health services are utilised by a multiracial population, language, beliefs, and cultural barriers might be frequent barriers in building effective communication between doctors and patients. To meet the expectation of the patients, the need for proper communication is vital in doctor–patient interactions.

### Strengths and Limitations

This is the first study in Fiji to undertake a mixed-method study of patient perceptions of doctor–patient communication. The sample size of the cross-sectional study exceeded the sample size in our power calculation, achieved with a response rate of 91%. We accessed patients whilst they were in health care centres so they could answer questions in relation to recent doctor–patient encounters. The addition of qualitative interviews added to the depth of our study. However, this study has some limitations in terms of international generalisability, particularly to higher-income countries. The majority of participants had higher-level education, and therefore, this might have affected their answers to the questions. The questionnaire used for the quantitative design was not tested for reliability before use, although it was calculated after the cross-sectional survey and was found to be both valid and reliable for future use. This qualitative study focused on the patients’ perception of doctors’ communication behaviour. Further study on doctors’ perceptions of factors affecting their communication is needed. This study was also limited to urban and peri-urban settings.

## 5. Conclusions

This study’s findings provide baseline information on the communication behaviour of doctors currently practicing in Suva, Fiji. It can be stated that currently, the majority of the patients perceived their doctor’s communication behaviour as fair in the Suva subdivision. Factors such as the doctor’s approach and tone, involving patients with patient-centred communication and care, providing advice, explaining their medical conditions, and explaining treatment being provided and probable prognosis in nonscientific language are important factors for doctors to practice while communicating with their patients. Given the benefits of better doctor–patient communication, such as better patient satisfaction, adherence to the treatment plan, better health outcomes, and less recurrent visits or admissions, it would be recommended for the policymakers in Fiji to implement refresher training on upgrading the communication skills of doctors every 2 to 3 years. It would be recommended that the concept of patient-centred care and communication be more prioritised in the medical curriculum whereby doctors’ communication skills are assessed together with their clinical skills.

### Implications for Public Health

Effective communication is needed in every relationship between the health care system and the public. Either in providing treatment to the patients or doing health promotion, effective communication can ensure better compliance with the plan. Health promotion programs will be more effective through effective communication processes.

## Figures and Tables

**Table 1:** Sociodemographic characteristics of participants (*n* = 375).

Characteristic	No. of Participants (*n*)	Percentage (%)
Age (mean ± SD)	38 ± 15 years	
18–50 years	293	78.1
>50 years	82	21.9
Gender		
Male	146	38.9
Female	229	61.1
Ethnicity		
i-Taukei	206	54.9
Indo-Fijian	141	37.6
Others	28	7.5
Education Level		
Higher	190	50.7
Secondary	153	40.8
Lower	32	8.5
Employment Status		
Studying	50	13.3
Working	216	57.6
Unemployed	37	9.9
Domestic Duties	72	19.2

**Table 2:** Participants’ response to communication-related statements.

Communication-Related Statements	Strongly Agree and Agree—*n* (%)	Not Sure *n* (%)	Strongly Disagree and Disagree—*n* (%)
1. Sometimes I feel the doctor ignores my concern. *	80 (23.3)	60 (16)	195 (62.7)
2. My doctor clearly explained to me what my medical condition was.	308 (81.1)	40 (10.7)	27 (7.2)
3. My doctor gave me opportunity to ask questions.	263 (70.1)	44 (11.7)	68 (18.2)
4. My doctor encouraged me to give my opinion about my medical treatment.	230 (61.1)	67 (17.9)	78 (20.8)
5. My doctor uses too many technical terms that confuse me. *	75 (20)	53 (14.1)	247 (65.9)
6. My doctor provided me as much information as I wanted.	269 (71.7)	55 (14.7)	51 (13.6)
7. My doctor treated me in a way that made me feel comfortable.	319 (85.1)	30 (8)	26 (6.9)
8. My doctor checked to ensure that I understood everything.	291 (77.6)	38 (10.1)	46 (12)

* Negative Statement.

**Table 3:** Distribution of participants based on levels of communication.

Communication Behaviour	Points Scale	*n* (%)	Mean (±SD)
Good	32–40	171 (45.6)	30 ± 5
Fair	17–31	201 (53.6)	
Poor	8–16	3 (0.8)	

**Table 4:** Characteristics of the participants (*n* = 20).

Variables	*n*	%
**Gender**		
Male	10	50
Female	10	50
**Race**		
i-Taukei	10	50
Indo-Fijian	10	50
**Age**		
<50 years	14	70
≥50 years	6	30

## Data Availability

Data can be found at the Open Science Framework (OSF): Doctor–Patient Communication in Fiji. DOI 10.17605/OSF.IO/9KXUM (Mohammadnezhad, 2020).
